# The Effect of COVID-19 Lockdown Among Adolescents with Type 1 Diabetes: A Systematic Review and Meta-Analysis

**DOI:** 10.2174/0115733998327893240905071326

**Published:** 2024-10-02

**Authors:** Aaliyah Momani, Aram Halimi, Seyed Saeed Hashemi Nazari, Zalikha Al-Marzouqi, Alireza Mosavi Jarrahi, Nabeel Al-Yateem, Syed Azizur Rahman, Amina Al-Marzouqi

**Affiliations:** 1 Maternal and Children Nursing Health Department, Faculty of Nursing, Applied Science Private University, 21 Al Arab St, Amman, Jordan;; 2 Research Center for Social Determinants of Health, Research Institute for Endocrine Sciences, Shahid Beheshti University of Medical Sciences, Tehran, Iran;; 3 Department of Epidemiology, School of Public Health and Safety, Shahid Beheshti University of Medical Sciences, Tehran, Iran;; 4 Prevention of Cardiovascular Disease Research Centre, Department of Epidemiology, School of Public Health and Safety, Shahid Beheshti University of Medical Sciences, Tehran, Iran;; 5 Maternal Health Department, Oman College of Health Sciences, North Batinah Branch, Sultanate of Oman, Muscat, Oman;; 6 Cancer Research Centre, Shahid Beheshti University of Medical Sciences, Tehran, Iran;; 7 College of Health Sciences, University of Sharjah, Sharjah, UAE

**Keywords:** Type 1 diabetes, adolescents, glycaemic control, COVID-19 pandemic, pre-lockdown period, post-lockdown period

## Abstract

**Objective:**

The aim of this study was to assess how the lockdown of the COVID-19 pandemic had affected the glycaemic control of adolescents aged 10-19 with type 1 diabetes.

**Methods:**

A comprehensive search of literature was performed in PubMed, Scopus, Web of Science, and ProQuest. Published articles up to September 2022 were included. The Glucose Monitoring Index (GMI) and HbA1c level were defined as outcome variables. Average glucose level was found to be a common variable in both HbA1c levels and GMI; therefore, HbA1c and GMI were converted to average glucose (mg/dL) using appropriate formulas. Studies reported the outcomes in two or three periods (pre-lockdown, lockdown, and post-lockdown) were included in the analysis. A paired wise meta-analysis was performed among the studies that reported all three periods. Homogeneity across studies was assessed using *I^2^* statistic.

**Results:**

Fourteen studies were included in the study. The pooled average glucose during the lockdown decreased to 166.9 mg/dL (95% CI, 153.78, 180.02) from 205.793 mg/dL (95% CI, 188.412, 223.173) during the pre-lockdown period, then it increased to 204.23 mg/dL (95% CI, 186.17, 222.29) during the post-lockdown period. A paired wise meta-analysis indicated a reduction in average glucose levels. However, it was not statistically significant, possibly due to the small number of studies that reported data from all three periods.

**Conclusion:**

Although the descriptive analysis of our study showed that the lockdown had affected (decreased) the average glucose level among adolescents with type 1 diabetes, this was not statistically significant in the pooled analysis.

## 1 INTRODUCTION

Type 1 diabetes is the most common endocrine chronic condition among adolescents. Over the past decades, every year its rate has been increasing by 3-5% globally [1]. In 2021, there were 8.4 million individuals with type 1 diabetes worldwide, of which 1.5 million were individuals under the age of 15 years [2]. According to the World Health Organisation (WHO), adolescence is a developmental period that involves individuals aged 10-19 years going through several developmental changes [3]. Adolescents with type 1 diabetes need to adhere to a strict and labour-intensive daily regimen to manage their condition, aiming to maintain optimal blood glucose levels [4]. Sub-optimal diabetes management can result in the development of both short- and long-term diabetes-related complications. These complications can affect the quality of life of adolescents and are associated with increased mortality [1]. Managing type 1 diabetes during adolescence can be challenging because of the various developmental processes happening at this stage; these include, but are not limited to brain, identity, cognitive, emotional, and other developments [5-7].

Glycaemic control among adolescents may be sub-optimal [8, 9]. For example, in one study of 4,499 adolescents aged 13-18 years conducted in the United States, only 32.4% of the participants achieved optimal glycaemic control (HbA1c level below 7% according to the American Diabetes Association recommendations for this age group) [9]. There are two explanations for the sub-optimal glycaemic control during adolescence. First is physiological, where hormonal changes during puberty can affect insulin sensitivity. Secondly, a combination of behavioural, psychosocial and emotional factors can affect diabetes management, hence glycaemic control [10-12]. Accordingly, it is important to understand type 1 diabetes management among adolescents, especially during global health crises.

One of the recent global health crises is the Coronavirus disease 2019 (COVID-19), which is an acute respiratory viral infection caused by SARS-CoV-2 that was first identified in December, 2019 in Wuhan, China [13]. In February 2020, COVID-19 rapidly spread to most countries in the world, and then it was declared a global pandemic [13]. All individuals at any age could be infected with the virus, and the infection could range from being asymptomatic to a severe form requiring intensive care treatments. Furthermore, individuals with underlying medical conditions such as diabetes, cancer, and chronic respiratory conditions were at higher risk of having the severe form of COVID-19, being hospitalised, and even die from the infection [14]. The incidence rates of COVID-19 among adolescents and children were lower than the incidence among adults [15]. Of the total global 4.4 million deaths due to COVID-19, 9,222 deaths occurred among adolescents [16]. Furthermore, diabetes mellitus is one of the most common morbidities found among patients with COVID-19 [17]. This indicates the importance of understanding how COVID-19 pandemic affects individuals with type 1 diabetes and its management.

COVID-19 pandemic and its associated restrictions may pose an additional risk for managing type 1 diabetes among adolescents whose glycaemic control during this period of their lives can be suboptimal, as discussed above. These restrictions can influence the daily routines of adolescents, their lifestyles, and the accessibility to healthcare services [18]. However, there are inconsistencies in the literature on how COVID-19 pandemic and its associated restrictions can affect the glycaemic control of adolescents with type 1 diabetes. Some studies reported decreased HbA1c (glycated haemoglobin) levels after the lockdown compared to pre-lockdown period [19, 20]. Other studies reported no changes in HbA1c levels [21]. Contrary to that, other studies indicated increased HbA1c levels [22]. Therefore, the aim of this systematic review and meta-analysis is to review the available literature on the topic of glycaemic control changes among adolescents aged 10-19 with type 1 diabetes during COVID-19 pandemic and to compare it to the pre-pandemic/ pre-lockdown levels.

## 2 MATERIALS AND METHODS

### 2.1 Data Sources and Searches

This systematic review and meta-analysis was conducted in accordance with Preferred Reporting Items for Systematic Reviews and Meta-Analyses (PRISMA) guidelines [23]. This protocol is registered on International Prospective Register for Systematic Reviews (PROSPERO) as CRD42022341726. Articles published in English dated from 2020 to September 2022 were included. Four databases were searched for relevant studies, including PubMed, Scopus, Web of Science, and ProQuest. The search strategy in all databases was as the following: on PubMed (glycaemic control[MeSH Terms]) OR (glycemic control), OR (blood glucose control), OR (glucose control), OR (blood glucose control), AND (type 1 diabetes[MeSH Terms]) OR (type 1 diabetes mellitus) OR (insulin-dependent) OR (insulin-dependent diabetes mellitus) OR (juvenile-onset diabetes mellitus) OR (IDDM) OR (diabetes mellitus sudden-onset) OR (diabetes mellitus sudden onset) AND (adolescent) OR (adolescence)) OR (teens)) OR (teen)) OR (teenagers)) OR (teenager)) OR (youth)) OR (youths)) OR (adolescent[MeSH Terms])). On Scopus (TITLE-ABS-KEY (adolescent OR adolescence OR teens OR teen OR teenagers OR teenager OR youth OR youths) AND TITLE-ABS-KEY ((type 1 diabetes) OR (type 1 diabetes AND mellitus) OR (insulin-dependent) OR (insulin-dependent AND diabetes AND mellitus) OR (juvenile-onset AND diabetes AND mellitus) OR (iddm) OR (diabetes AND mellitus AND sudden-onset) OR (diabetes AND mellitus AND sudden AND onset)) AND TITLE-ABS-KEY ((glycaemic AND control) OR (glycemic AND control) OR (blood AND glucose AND control) OR (glucose AND control) OR (blood AND glucose AND control))). On Web of Science ((TS=(adolescent OR adolescence OR teens OR teen OR teenagers OR teenager OR youth OR youths)) AND TS=((type 1 diabetes) OR (type 1 diabetes AND mellitus) OR (insulin-dependent) OR (insulin-dependent AND diabetes AND mellitus) OR (juvenile-onset AND diabetes AND mellitus) OR (iddm) OR (diabetes AND mellitus AND sudden-onset) OR (diabetes AND mellitus AND sudden AND onset))) AND TS=((glycaemic AND control) OR (glycemic AND control) OR (blood AND glucose AND control) OR (glucose AND control) OR (blood AND glucose AND control)). And on ProQuest ab(adolescent OR adolescence OR teens OR teen OR teenagers OR teenager OR youth OR youths) AND ab((type 1 diabetes) OR (type 1 diabetes AND mellitus) OR (insulin-dependent) OR (insulin-dependent AND diabetes AND mellitus) OR (juvenile-onset AND diabetes AND mellitus) OR (iddm) OR (diabetes AND mellitus AND sudden-onset) OR (diabetes AND mellitus AND sudden AND onset)) AND ab((glycaemic AND control) OR (glycemic AND control) OR (blood AND glucose AND control) OR (glucose AND control) OR (blood AND glucose AND control)). EndNote software was used to manage retrieved articles.

### 2.2 Inclusion and Exclusion Criteria

Studies for this systematic review and meta-analysis were selected according to the following criteria: no limitations by the sort of setting, country, or study design were imposed on the search. Studies examining adolescents (aged 10-19 years, according to WHO definition of adolescents [3]) were included, studies of both children / young adult populations in addition to adolescents were included. Moreover, studies of adolescents with type 1 and type 2 diabetes were included only if the data were presented separately. If data on adolescents were not presented separately, the corresponding author was contacted to request relevant data *via* email. In case of no response within a month of correspondence, the analysis was performed using available data or the article was excluded from this meta-analysis. The exclusion criteria included letters to the editor, duplicate studies, systematic review and meta-analysis studies, and studies where it was not possible to extract means and SDs for relevant outcomes.

### 2.3 Data Extraction and Quality Assessment

Titles and abstracts yielded by database search were screened by two reviewers independently. All studies that seemed to have met the inclusion criteria based on the initial screening were obtained in full text and then screened again against the inclusion criteria. EndNote software was used during this stage. The reviewers worked independently and met on a regular basis to discuss any disagreements or issues throughout the process. Microsoft Excel was used to develop a specific data extraction form for this meta-analysis. The extraction form included the following information: full reference, country/ setting, design, participants, sampling, data collection, data analysis, period of data collection, HbA1c levels before the pandemic, HbA1c levels during the pandemic, other results, critical appraisal, notes, extractor name, and extraction date. The reviewers had a discussion before the data extraction process begun to discuss the level of needed detail during the extraction process. Any data extraction discrepancies were resolved through discussions. Joanna Briggs Institute (JBI) critical appraisal tools [24] were used to assess the quality of studies and address the possibility of bias (Supplementary Materials Table **A**).

### 2.4 Data Analysis and Synthesis

A narrative summary of each included study in this systematic review and meta-analysis was presented in a table. When data extraction started, we realized that some studies reported Glucose Monitoring Index (GMI) with standard deviation. After reviewing the literature, average glucose level was found as a common variable in both HbA1c levels and GMI [25; 26]; therefore, HbA1c and GMI were converted to average glucose (mg/dL) using these formulas: (HbA1c*35.6)-77.3 and (GMI-3.31)/0.02392, respectively. In addition, standard deviation (SD was calculated using these formulas: SD_AG from HbA1c_ = 35.6*SD_HbA1c_, SD_AG from GMI_ = SD_GMI_/0.02392, respectively [25, 26].

Homogeneity across included studies was assessed using *I*^2^ statistic [24], and a meta-analysis would only be performed if there was sufficient homogeneity. *I*^2^ statistic of 0-40%: might not be important; 30-60%: may represent moderate heterogeneity; 50-90%: may represent substantial heterogeneity; 75-100%: considerable heterogeneity [27]. The absolute amount of heterogeneity would also be measured using tau square. A Funnel plot would be used to assess publication bias. In case of substantive heterogeneity, meta-regression or subgroup analysis would be used whenever possible.

## 3 RESULTS

### 3.1 Study Selection

Study selection process is presented in Fig. (**1**). The total number of articles that were identified from the databases after excluding duplicate articles was 2008, and 1955 articles were excluded after titles and abstracts were scanned. Only 53 articles were considered for full-text reading. Furthermore, 35 articles were excluded because the effect of COVID-19 lockdown among adolescents with type 1 diabetes was not assessed, and 4 articles were excluded because the confidence interval was not mentioned.

In total, 14 retrospective articles were eligible for this meta-analysis. The characteristics and the quality of included studies in this meta-analysis are presented in Table **1**. The data were analysed using STATA 17 software.

### 3.2 Study Characteristics

In total, 898 patients from eight countries were included in this meta-analysis. The sample size of included articles was variable, it ranged from 13 to 194 participants.

The results of this systematic review and meta-analysis are presented in six sections: pre-lockdown period; lockdown period; post-lockdown period; pre-lockdown compared to lockdown period; lockdown compared to post-lockdown period; and pre-lockdown compared to post-lockdown period. We decided to present the results in these six sections based on the row data from included studies, meaning that there were wide variations between studies in the lockdown periods because of the different measures (and periods) taken by each country during the pandemic. Nevertheless, to facilitate the analysis and the presentation of the results, we decided to present the results in three periods (pre-lockdown, lockdown and post-lockdown) and then make a comparison between these periods regardless the actual duration of the lockdown period in each country. A descriptive analysis of the average glucose levels in each period is presented. The pre-lockdown period is the period before the pandemic was declared. The post-lockdown period is the period after the lockdown measures were eased based on each country’s measures.

### 3.3 Pre-Lockdown Period

Fig. (**2**) shows the pooled results of seven articles that reported data during the pre-lockdown period, the heterogeneity using *I^2^* was 93.35; therefore, a random-effects model was used. The effect size of the average glucose level during the pre-lockdown period was 205.793 mg/dL (95% CI, 188.412, 223.173).

### 3.4 Lockdown Period

Fig. (**3**) shows the pooled results from seven articles that reported data during the lockdown period, the heterogeneity using *I^2^* was 96.36; therefore, a random-effects model was used. The effect size of the average glucose level during the lockdown period was 166.9 mg/dL (95% CI, 153.78, 180.02).

### 3.5 Post-Lockdown Period

Fig. (**4**) shows the pooled results from 10 articles that reported data during the post-lockdown period; the heterogeneity using *I^2^* was 97.94; therefore, a random-effects model was used. The effect size of the average glucose level during the post-lockdown period was 204.23 mg/dL (95% CI, 186.17, 222.29).

According to what is shown in the pooled effect size of average glucose levels in the pre-lockdown, lockdown, and post-lockdown periods, which were 223.58, 166.9, 204.23, respectively, this shows that the average glucose during the lockdown period was lower than the other two periods.

### 3.6 Pre-lockdown Period Compared to Lockdown Period

Fig. (**5**) shows the pooled results from five articles that reported data during the pre-lockdown and the lockdown periods; the heterogeneity using *I^2^* was 92.5%; therefore, a random-effects model was used, Chi^2^ was used to test homogeneity, it was 88.21, *P* value > 0.000. The pooled effect size was 0.48 (95% CI, -0.4, 1.4); this shows that there was not a significant difference in the average glucose levels between these two periods. It means that the lockdown did not have an effect on glycaemic control among adolescents with type 1 diabetes.

### 3.7 Lockdown Period Compared to Post-lockdown Period

Fig. (**6**) shows the pooled results from three articles that reported data during the lockdown and the post-lockdown periods, the heterogeneity using *I^2^* was 00.0%, and Chi^2^ was 1.27, *P* value > 0.7364. Therefore, there was no heterogeneity between studies and a random-effects model was used. The pooled effect size was 0.14 (95% CI, -0.03, 0.31); this shows that there was not a significant difference in glycaemic control between these two periods.

### 3.8 Pre-lockdown Period Compared to Post-lockdown Period

Fig. (**7**) shows the pooled results from eight articles that reported data during the pre-lockdown and the post-lockdown periods. The heterogeneity *I^2^* was 27.26%, and Chi^2^ was used to test homogeneity, it was 13.06, *P* value > 0.2202. Therefore, there was no heterogeneity between studies and random-effects model was used. The pooled effect size was 0.06 (95% CI, -0.1, 0.22); this shows that there was not a significant difference in average glucose levels between these two periods. It means that the pandemic/ lockdown did not have an effect on glycaemic control among adolescents with type 1 diabetes.

### 3.9 Heterogeneity

The observed heterogeneity among the included studies is indeed significant, as reflected in the high I^2^ values across different periods (*e.g*., *I^2^* = 97.94% during the post-lockdown period). This high heterogeneity suggests considerable variability in the effects reported by different studies, which could be attributed to differences in study designs, populations, and contexts.

The substantial heterogeneity implies that while we can derive an overall trend, the individual study results may not be directly comparable. This variability can influence the generalizability of our findings. For example, the variation in average glucose levels across studies suggests that factors such as geographical location, healthcare access, and adherence to diabetes management protocols during the lockdown periods might have significantly influenced the outcomes. Understanding these variations is crucial for tailoring interventions to specific contexts rather than applying a one-size-fits-all approach.

#### 3.9.1 Publication Bias

In this study, publication bias was assessed using funnel plots, with results presented in three distinct sections: pre-lockdown compared to the lockdown phase, lockdown compared to the post-lockdown phase and pre-lockdown compared to the post-lockdown phase.

##### 3.9.1.1 Publication Bias for Pre-Lockdown Phase Compared to Lockdown Phase

As depicted in Funnel Plot 1 (Supplementary Materials Fig. **S1**), our analysis indicates the presence of publication bias, suggesting the potential exclusion of some studies from publication during this phase.

##### 3.9.1.2 Publication Bias for Lockdown Phase Compared to the Post-Lockdown Phase

Funnel Plot 2 (Supplementary Materials Fig. **S2**) also reveals evidence of publication bias during the lockdown phase compared to the post-lockdown phase, which may be attributed to the lack of publication of certain studies.

##### 3.9.1.3 Publication Bias for Pre-Lockdown Phase Compared to Post-Lockdown Phase

Similarly, Funnel Plot 3 (Supplementary Materials Fig. **S3**) illustrates the presence of publication bias when comparing the pre-lockdown phase to the post-lockdown phase due to potential omission of specific studies.

## 4 DISCUSSION

This systematic review and meta-analysis included 14 studies from different countries that aimed at exploring the influence of COVID-19 lockdown on glycaemic control among adolescents aged 10-19 years diagnosed with type 1 diabetes. Based on what was reported in the included studies, the results were presented in six sections that compared different periods: pre-lockdown; lockdown; and post-lockdown periods. When comparing these periods: pre-lockdown and lockdown; lockdown and post-lockdown; and pre-lockdown and post-lockdown, the pooled results showed that, on one hand, the lockdown had decreased the average glucose levels among adolescent with type 1 diabetes, this could be explained by that during the lockdown periods adolescents could concentrate more on diabetes management away from factors (such as managing the condition away from home) that could influence diabetes management, this was reflected on their glycaemic control. On the other hand, as the lookdown ceased and relaxed, the metrics headed back toward their pre-lockdown values; however, this was not statistically significant in the pooled analysis, possibly due to a small number of studies.

The pooled results in this systematic review and meta-analysis were not consistent with some individual studies that were included in this meta-analysis. On the one hand, some studies showed that glycaemic control did not change during the COVID-19 pandemic lockdown [30]. On the other hand, other studies showed that glycaemic control during the COVID-19 pandemic lockdown was suboptimal compared to the glycaemic control before the pandemic [28, 29]. Whereas, other studies showed mixed results.

Individuals with diabetes have more severe forms of COVID-19 [41, 42], and, in turn, hyperglycaemia is associated with the severity of COVID-19 symptoms [19]. Moreover, individuals with COVID-19 are prone to develop a new onset of diabetes [41, 42]. COVID-19 pandemic is associated with restriction measures such as lockdowns, which may pose a risk for managing type 1 diabetes among adolescents. This means that the daily routines of adolescents, their lifestyle, and their accessibility to healthcare services might have been influenced by the lockdown [18]. For instance, changes in adolescents’ diet, schooling, and activity levels, can affect their health-related quality of life [43, 44]. In addition to that, lockdowns had affected adolescents’ mental well-being. For example, some of the adolescents experienced fear, discouragement, uncertainty, and anxiety during the lockdown periods [45, 46]. Also, technology had played an important role during lockdown periods [45]. This shows the complexity of type 1 diabetes management during lockdown periods. Therefore, it is important to consider different factors that can influence diabetes management and glycaemic control among adolescents with type 1 diabetes during lockdown periods.

It is important to indicate that several studies that were included in this systematic review and meta-analysis aimed to measure the change in glycaemic control before, during, and /or after the COVID-19 pandemic lockdown alongside other factors that could possibly influence glycaemic control. For example, one study investigated eating habits during the pandemic and how the glycaemic control changed among adolescents with type 1 diabetes [30]. Another study explored the change in glycaemic control during COVID-19 pandemic lockdown along with other factors such as insulin type, physical activities, and the number of telemedicine visits [32]. Moreover, one study explored the relationship between anxiety, depression, and glycaemic control during lockdowns [37]. Furthermore, COVID-19 pandemic lockdowns resulted in shifting diabetes consultation from face-to-face to other virtual means such as telephone or video calls. This rapid shift in utilising telemedicine during the COVID-19 pandemic could have also had an effect on glycaemic control of adolescents with type 1 diabetes [31]. Lastly, the type of insulin taken and the route of its administration (using injections or pumps) might have also influenced glycaemic control during the pandemic [28, 29, 36, 37]. All the above-mentioned factors, alongside COVID-19 pandemic lockdown, may influence glycaemic control among adolescents with type 1 diabetes; however, they were not taken into account when this systematic review and meta-analysis was conducted as it was beyond the aim of this study. It is suggested to use the “syndemic” approach to improve the quality of care provided to individuals with diabetes and other chronic conditions, as this approach emphasises the relevance of biological, social, environmental and economic aspects that influence the health of individuals with chronic conditions [41]. All this shows the complexity of type 1 diabetes management as several factors can influence glycaemic control.

## 5 STRENGTHS AND LIMITATIONS

This is the first systematic review and meta-analysis that focuses on a specific age group which is adolescents aged 10-19 years and diagnosed with type 1 diabetes that had analysed the change in glycaemic control before, during and after COVID-19 lockdowns from different countries. The researchers acknowledge that more research has been published on this topic since conducting our database search, which can be considered in future reviews and meta-analyses. Moreover, with the available data, adolescents from different settings than the ones included in this study may have different glycaemic controls before, during, and after the lockdown periods; therefore, this should be considered when generalising the results of this study. Furthermore, this systematic review and meta-analysis included adolescents with type 1 diabetes only, so, adolescents with type 2 diabetes may have different glycaemic controls before, during, and after lockdown periods.

## CONCLUSION

The descriptive analysis of our study showed that the lockdown had decreased the average glucose level among adolescents with type 1 diabetes; however, this was not statistically significant as the pooled analysis of the included studies, this systematic review and meta-analysis showed that COVID-19 lockdown did influence the average glucose among adolescents diagnosed with type 1 diabetes. This can help in understanding how lockdowns affected individuals with type 1 diabetes in terms of their glycaemic control. Furthermore, this may help in understanding how a future medical emergency may influence glycaemic control among adolescents with type 1 diabetes.

## RECOMMENDATIONS

Future research, specifically meta-analyses, should be conducted with a focus on more recent studies on the topic of glycaemic control among adolescents before and during the pandemic that were published after this meta-analysis was conducted. Moreover, more research is needed to explore how changes in daily routines and lifestyle during the lockdown/ pandemic could have influenced type 1 diabetes management among adolescents. Furthermore, exploring the underlying reasons for changes in glucose levels during the lockdown period warrants further research to improve diabetes management. Lastly, exploring changes in glycaemic control before and during lockdown/ pandemic among adolescents with type 2 diabetes should be considered in future studies and reviews.

## Figures and Tables

**Fig. (1):**
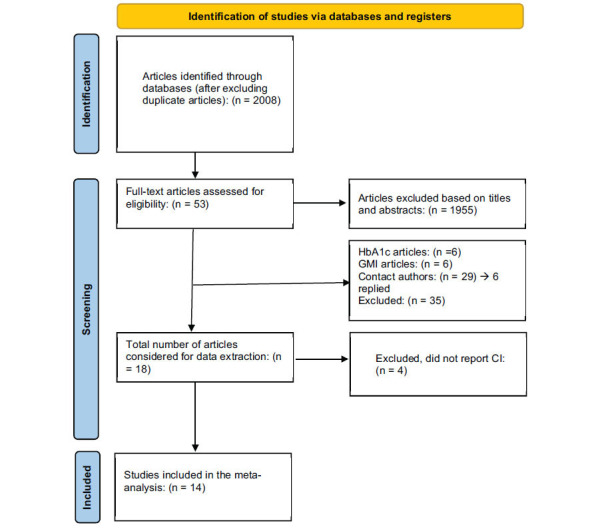
Flowchart of studies selection.

**Fig. (2):**
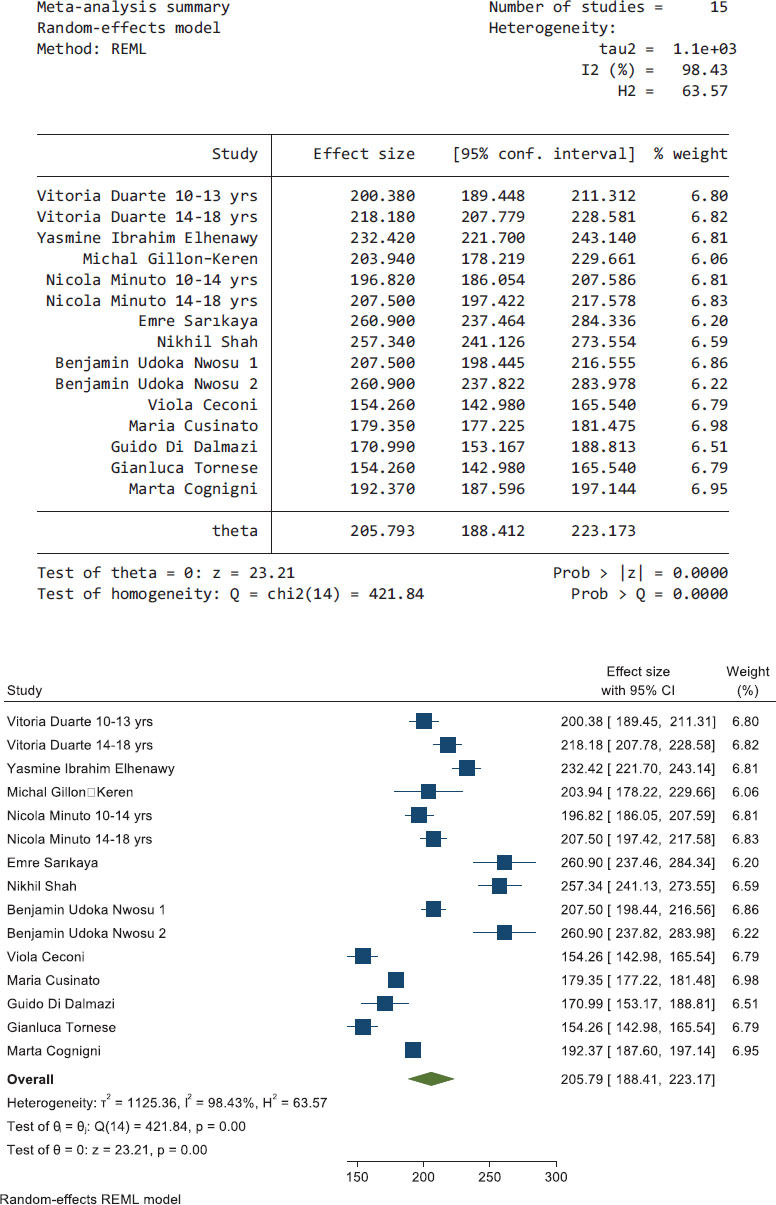
The pooled results from seven articles and their forest plot. The pooled average glucose is 205.793 mg/dL (95% CI, 188.412, 223.173).

**Fig. (3):**
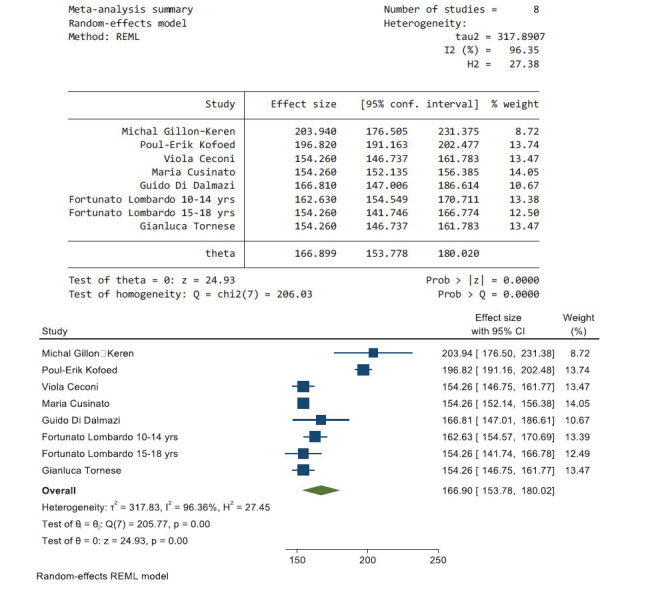
The pooled results from seven articles and their forest plot. The pooled average glucose is 166.9 mg/dL (95% CI, 153.78, 180.02).

**Fig. (4):**
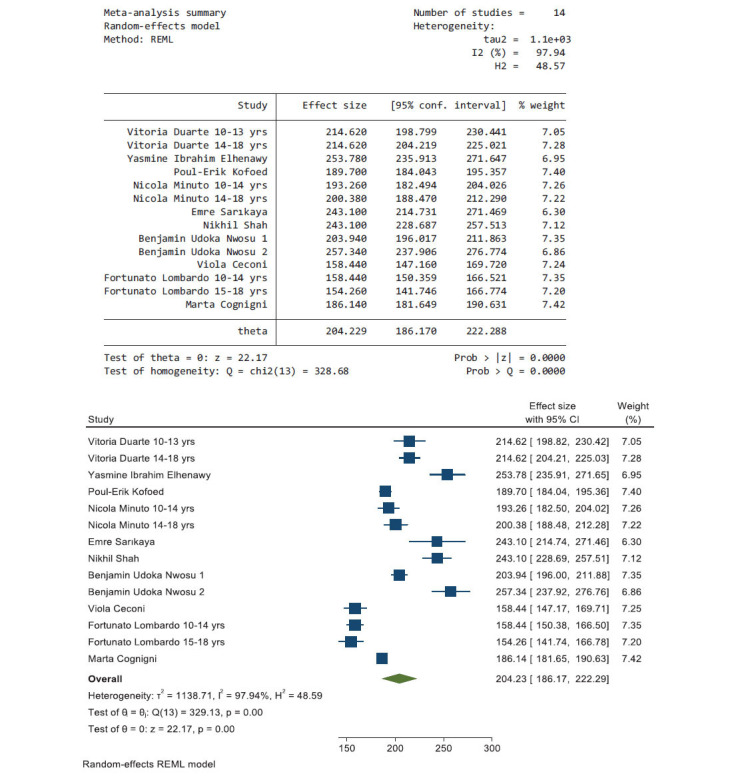
The pooled results from 10 articles and their forest plot. The pooled average glucose is 204.23 mg/dL (95% CI, 186.17, 222.29).

**Fig. (5):**
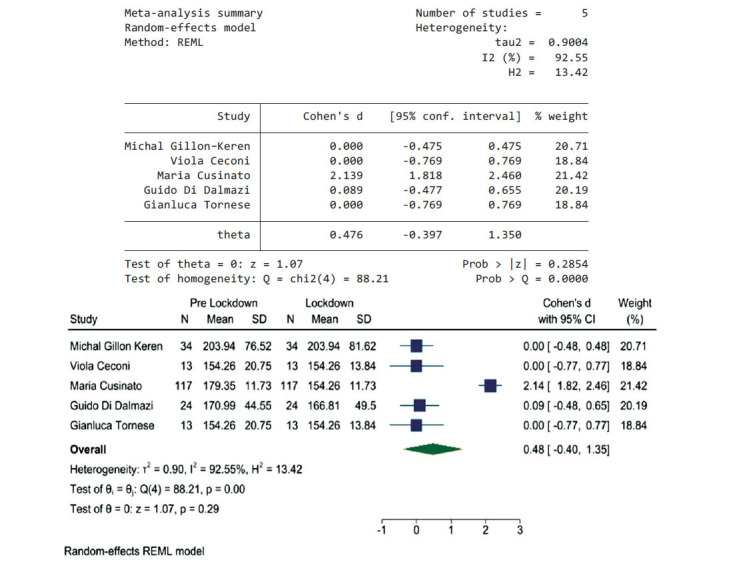
The pooled results from five articles and their forest plot. The pooled average glucose is 0.48 (95% CI, -0.4, 1.35).

**Fig. (6):**
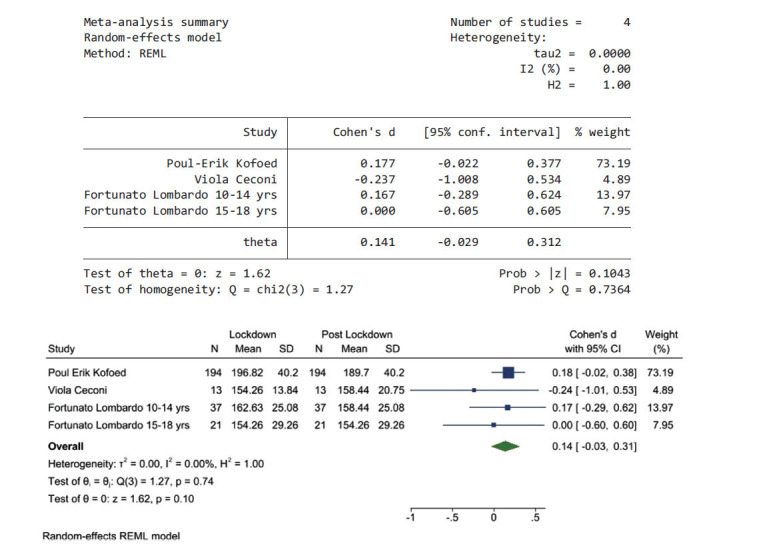
The pooled results from three articles and their forest plot. The pooled average glucose is 0.14 mg/dL (95% CI, -0.03, 0.31).

**Fig. (7):**
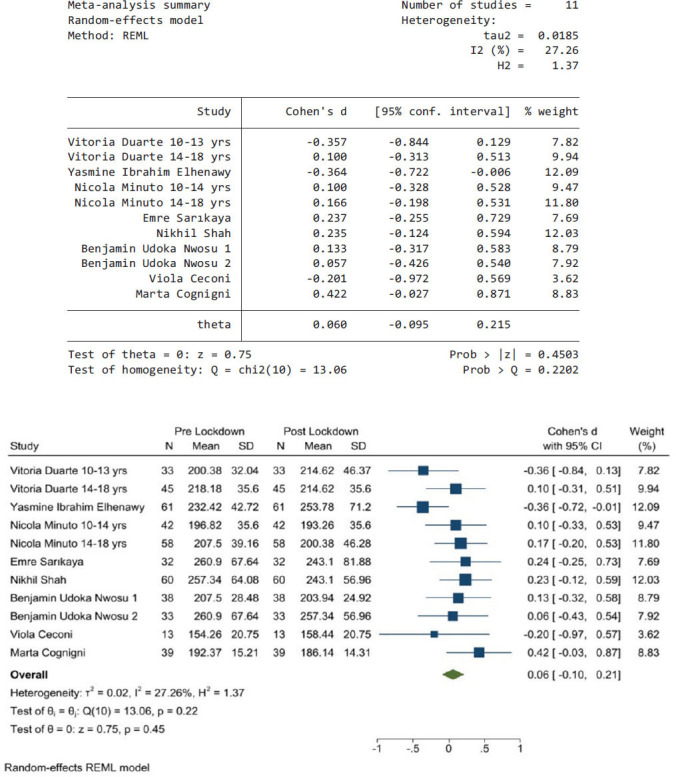
The pooled results from eight articles and their forest plot. The pooled average glucose is 0.06 mg/dL (95% CI, -0.1, 0.21).

**Table 1:** A summary of extracted data from 14 articles included in the meta-analysis.

**Study Name**	**Sample Size**	**Country**	**Quality**	**Pre-Lockdown Period Average glucose mg/dL**	**Standard deviation Pre-Lockdown Period**	**Lockdown Period Average Glucose mg/dL**	**Standard Deviation Lockdown Period**	**Post-Lockdown Period** **Average Glucose mg/dL**	**Standard Deviation Post-Lockdown Period**
Vitoria Duarte 10-13 years old [28]	33	Portugal	High	200.38	32.04	N/A	N/A	214.62	46.37
Vitoria Duarte 14-18 years old	45	Portugal	High	218.18	35.60	N/A	N/A	214.62	35.60
Yasmine Ibrahim Elhenawy [29]	61	Egypt	High	232.42	42.72	N/A	N/A	253.78	71.20
Michal Gillon-Keren [30]	34	Israel	High	203.94	76.51	203.94	81.62	N/A	N/A
Poul-Erik Kofoed [31]	194	Denmark	High	N/A	N/A	196.82	40.20	189.70	40.20
Nicola Minuto 10-14 years old [32]	42	Italy	High	196.82	35.60	N/A	N/A	193.26	35.60
Nicola Minuto 14-18 years old	58	Italy	High	207.50	39.16	N/A	N/A	200.38	46.28
Emre Sarıkaya [33]	32	Turkey	High	260.90	67.64	N/A	N/A	243.10	81.88
Nikhil Shah [34]	60	India	High	257.34	64.10	N/A	N/A	243.10	56.96
Benjamin Udoka Nwosu 1 [35]	38	USA	High	207.50	28.48	N/A	N/A	203.94	24.92
Benjamin Udoka Nwosu 2	33	USA	High	260.90	67.64	N/A	N/A	257.34	56.96
Viola Ceconi [36]	13	Italy	Moderate	154.26	20.75	154.26	13.84	158.44	20.75
Maria Cusinato [37]	117	Italy	High	179.35	11.73	154.26	11.73	N/A	N/A
Guido Di Dalmazi [38]	24	Italy	High	170.99	44.55	166.81	49.50	N/A	N/A
Fortunato Lombardo 10-14 years old [39]	37	Italy	Moderate	N/A	N/A	162.63	25.08	158.44	25.08
Fortunato Lombardo 15-18 years old	21	Italy	Moderate	N/A	N/A	154.26	29.26	154.26	29.26
Gianluca Tornese [40]	13	Italy	High	154.26	20.75	154.26	13.84	N/A	N/A
Marta Cognigni [19]	39	India	High	192.37	15.21	N/A	N/A	186.14	14.31

## Data Availability

All data generated or analysed during this study are included in this published article.

## LIST OF ABBREVIATIONS

GMI: Glucose Monitoring Index
JBI: Joanna Briggs Institute
WHO: World Health Organisation

## STANDARDS OF REPORTING

PRISMA guidelines and methodology were followed.

## SUPPLEMENTARY MATERIAL

PRISMA checklist is available as supplementary material. Supplementary material is available on the publisher's website along with the published article.
